# Experimental Investigation on the Use of Selenice Natural Bitumen as an Additive for Pavement Materials

**DOI:** 10.3390/ma14041023

**Published:** 2021-02-21

**Authors:** Chichun Hu, Yucan Mai, Augusto Cannone Falchetto, Edith Tartari

**Affiliations:** 1 School of Civil Engineering and Transportation, South China University of Technology, Guangzhou 510006, China; cthu@scut.edu.cn (C.H.); ctyucanmai@mail.scut.edu.cn (Y.M.); 2 Department of Civil & Environmental Engineering, University of Alaska Fairbanks, Fairbanks, AK 99775, USA; 3 Department of Civil Engineering, Aalto University, 02150 Espoo, Finland; 4 R&D Department, Selenice Bitumi SH.A., 1001 Tirana, Albania; e.tartari@selenicebitumi.com

**Keywords:** natural asphalt binder, asphalt mixture, rheological characterization, mechanical performance, modification mechanism

## Abstract

As a good asphalt modifier, natural asphalt has been the focus of more attention because of its low price and ability to improve the performance of modified asphalt. In this paper, the incorporation of a natural asphalt binder in the production of bituminous materials for pavement application in China was experimentally investigated to evaluate the feasibility of such a process and its potential benefits in terms of performance. For this purpose, an asphalt binder conventionally used in the south of China was blended with various percentages of a hard natural binder obtained from the region of Selenice in Albania. The content of Selenice natural bitumen (SNB) was 80.5%, having high molecular weight and the advantages of good stability and compatibility with virgin asphalt. The physical, rheological, and mechanical properties, as well as the modification mechanism of the binder and corresponding asphalt mixture, were evaluated in the laboratory. It was observed that the hard binder improved the response of the binder blend at high and intermediate temperature; this reflected a better stability, improved moisture susceptibility, and enhanced rutting resistance of the mixture. Fluorescence microscopy showed that after dissolving, the size of the SNB modifier became smaller and its distribution was uneven, presenting three forms, granular, agglomerated, and flocculent properties. Chemical test results showed that the modification mechanism of SNB was mainly related to the enhancement of hydrogen bonds and Van der Waals forces caused by sulfoxide and carbonyl along with the stress concentration caused by silica particles. Molecular composition revealed that the proportion of middle molecules has reduced while the proportion of large molecules has increased. It is considered that SNB is a promising low-priced natural modifier with excellent rutting resistance properties. Future research will be focused on the economic analysis, pavement life cycle assessment of SNB modified asphalt, and its application in perpetual pavements.

## 1. Introduction

Asphalt mixture is the most common material used for pavement applications. Conventionally, this consists of a composite containing filler and aggregate bound together by a matrix of asphalt binder. Mix design is performed to achieve specific mixture characteristics so that the final product can withstand the stresses induced by traffic loading and temperature variation, as well as the action of the climate and environment where the pavement is constructed. When the required performance is not met by the based materials, different additives and components are incorporated into the asphalt mixture. This includes polymer modifiers, fibers, oxidants, antioxidants, and anti-stripping agents [1,2,3,4,5]. In addition, when reclaimed asphalt pavement (RAP) is used, rejuvenating agents [6] and soft binders [7] may be selected to mitigate the effect of the hard and oxidized RAP binder on the material performance.

Research performed on flexible pavements that exhibited a long service life has shown that there are substantial advantages in the design and use of asphalt mixtures with very high modulus or high modulus asphalt (HiMA) [8]. This type of mixture was originally developed in the 1980s by the Laboratoire Central des Ponts et Chaussées (LCPC) [9]. Over the years, several studies were conducted on the use of HiMA, in Europe [10] and South Korea [11], observing that binder source is critical for preparing asphalt mixture with a high modulus. Laboratory experimentation combined with finite element analysis was conducted in China to evaluate the use of high modulus asphalt mixture, showing improvement for permanent deformation and a reduction in shear strain [12,13]. A more comprehensive research effort performed in the United States demonstrated that high modulus mixtures can improve the pavement design performance in terms of rutting, fatigue cracking, and ride quality, although further validation is recommended [8].

In view of the increase in the use of high modulus asphalt mixture in pavement construction, there is a need for hard bitumen. While different techniques such as blowing process or vacuum distillation and propane-precipitated asphalt [9] can be used to achieve the desired gradation, blending a soft binder with a natural binder additive represents an alternative option. For example, the natural bitumen originating from the region of Selenice in Albania has found considerable application in different pavement-related constructions [14], to prepare hard asphalt binders to be used in the design of mixtures. This natural bitumen is commonly found in the cracks of rocks and presents a certain degree of impurity mainly consisting of fine particles. Selenice deposits show geomorphological variability and complex geological genesis. This is due to the combined effect of heat, pressure, oxidation, and bacteria, resulting in a hard asphalt material identified as Selenice natural bitumen (SNB) in this paper.

In a previous study [15], the use of such a natural additive resulted in a decrease in penetration and an increase in softening point. Tartari (2018) reported that when incorporating 8–10% of this hard natural additive, a decrease in one penetration grade could be experienced depending on the original asphalt binder selected for the blending process [14]. The use of the natural binder was also found to be beneficial for aging phenomena. The impact of the laboratory aging on the material response was less consistent for the modified binder than for the original, suggesting that the natural binder could work as an aging inhibitor [14,16].

Not only SNB but also other kinds of natural asphalts have found broad application and scientific research prospects. Xinjiang rock asphalt (XRA) has been proved to have good high temperature performance, with an optimal best dosage of 12 wt.% [17]. A different study has demonstrated that Qingchuan rock asphalt (QRA) can reduce the temperature sensitivity, improve the water stability, fatigue resistance, and the high temperature stability of asphalt and asphalt mixture [18]. Economic analysis of previous studies shows that Buton rock asphalt (BRA) has high economic and environmental value, as well as being less expensive than SBS modified asphalt [19,20]. The physical, chemical, and rheological properties of Buton rock asphalt (BRA) modified asphalt and asphalt mixture, have also been widely studied. The results of different experiments showed that BRA subjected to physical mixing revealed an improved softening point, and reduced penetration. In addition, it was observed that BRA modified asphalt prepared through the wet process presents a better performance in comparison to the corresponding material fabricated with the dry process [21,22,23,24]. The relationship between the microstructure and the performance of BRA modified asphalt was investigated by using surface free energy and an infrared spectrum analysis [25]. A new method of dry mixing and weighing was presented to evaluate the adhesion between BRA modified asphalt and coarse aggregate, with a recommended preheating temperature of 90–96 °C [26]. Li et al. (2020) used three different kinds of rock asphalt to modify virgin asphalt. They observed that the self-healing performance of Qingchuan rock asphalt was better than that of Uintaite modifier and Buton rock asphalt [27]. Other efforts showed that refined BRA can also be used for thin layer asphalt pavement [28]. BRA has also been combined with various modifiers, such as bio-oil, styrene–butadiene rubber, and other rock asphalts to modify the asphalt binder [29,30,31].

Like other kinds of natural asphalts, SNB also has significant potential for application with remarkable engineering benefits. SNB modifiers can replace expensive polymer modifiers because of its low price. The existing literature has proved the feasibility of SNB modifier application, but there is a lack of in-depth research on the modification mechanism of SNB modified asphalt, as well as comprehensive and systematic research on the physical, conventional, chemical, rheological, and mechanical performance of both SNB modified asphalt and its corresponding mixture.

## 2. Objective and Research Approach

**Figure 1:**
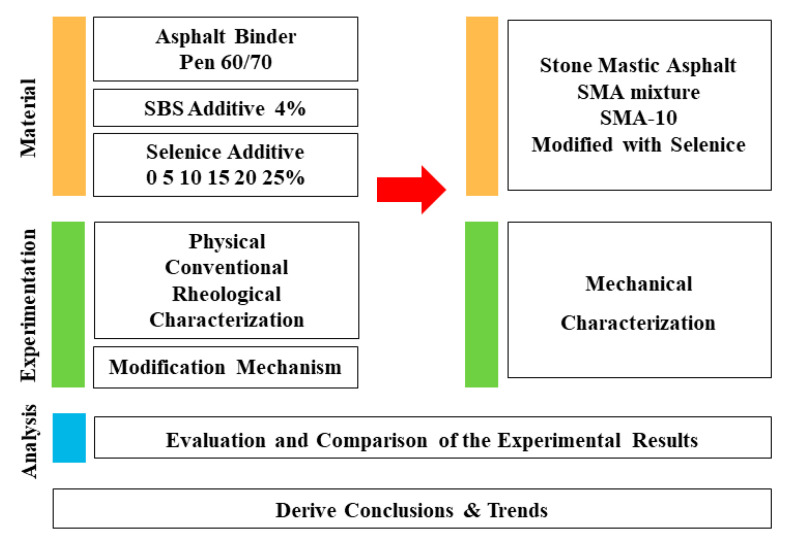
Schematic of the research approach.

In this paper, the feasibility of using the refined Selenice natural bitumen as an asphalt binder additive in China is investigated. Firstly, the impact of the SNB additive is evaluated at the binder level and then, based on the results obtained, the mechanical behavior of an SNB modified stone mastic asphalt (SMA) mixture is explored. To achieve this goal, a comprehensive set of laboratory tests was performed. This includes the determination of physical properties of the material; the evaluation of the conventional and rheological behavior as well as the modification mechanism of a common paving asphalt binder having penetration grade of 60/70 (Pen 60/70) (JTG E20 T0604-2011) [32] with SNB additive at five different percentages; and the estimation of the mechanical response of an SMA-10 prepared with the addition of SNB and presenting a nominal maximum aggregate size (NMAS) of 10 mm. Figure 1 presents the research approach adopted in the present study.

In order to achieve this goal, a series of tests were performed on SNB modified asphalt binder. This included physical, empirical, rheological-frequency and temperature sweep (JTG E20 T0628-2011) [33], multiple stress creep recovery (MSCR) (AASHTO T350-2019) [34], zero shear viscosity (ZSV), linear amplitude sweep (LAS) (AASHTO TP101-2014) [35], bending beam rheometer (BBR) (JTG E20 T0627-2011) [36], rotational viscosity (JTG E20 T0625-2011) [37] methods; as well as the chemical experimental methods of fluorescence microscopy (FM, Olympus Corporation, East Sussex, UK), Fourier-transform infrared spectroscopy (FTIR, Bruker Corporation, Karlsruhe, Germany), and gel permeation chromatography (GPC, Malvern Corporation, Worcestershire, UK). In addition, a Marshall test (JTG E20 T0709-2011) [38], indirect tensile strength (ITS) test (JTG E20 T0729-2011) [39], and Rutting test (JTG E20 T0719-2011) [40] were conducted to evaluate the mechanical properties of SNB modified asphalt mixture. This paper is intended to provide extensive and thorough research guidance on the application and future research of modified asphalt by the SNB.

## 3. Investigation at the Asphalt Binder Level

This section presents the experimental work and analysis performed on the SNB and on the blend of asphalt binder and SNB which was used to design the specific SNB modified asphalt mixture (see Section 4).

### 3.1. Preparation of the SNB Modified Asphalt Binder

A virgin asphalt binder commonly adopted for paving purposes in the south of China was selected for the present research as reference material. The binder, which presented a penetration grade of 60/70 (Pen 60/70) (JTG E20 T0604-2011) [32], was initially characterized (Table 1) and then used to prepare SNB modified binder blends.

**Figure 2:**
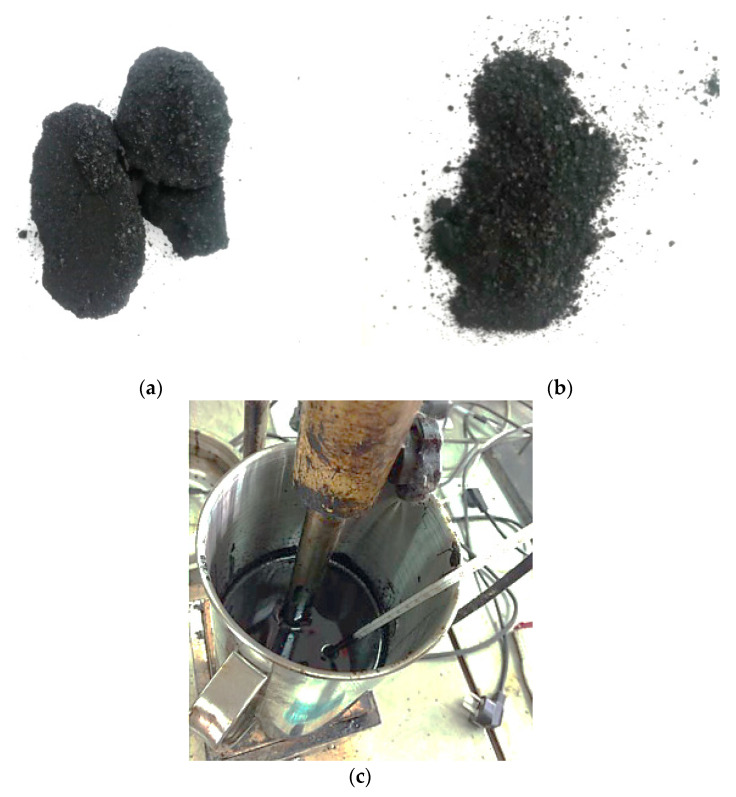
Preparation process: (**a**) Selenice natural bitumen (SNB); (**b**) SNB additive; (**c**) mixing process.

The blocks and agglomeration of SNB (Figure 2a) were first reduced to finer material (Figure 2b) through a grinding and crushing process. The size of finer SNB particles was roughly 1 mm (in the range of mesh 18) and the moisture content of SNB in its natural state was 2.46%. Then, the SNB in its natural state was preheated to remove the moisture in the binder blends to be prepared with the Pen 60/70 binder. Blending was initially performed with a glass rod for 15 min at a temperature of 165 °C, followed by the action of a high shear blending at 10,000 rpm for an additional 45 min (Figure 2c). SNB was added to the blend at different percentages: 0% (control), 5%, 10%, 15%, 20% and 25% by weight of SNB modified asphalt binder. Each batch of blend consisted of approximately 1.2 kg of SNB modified binder.

In order to better explain the performance of SNB modified asphalt, an additional control material group, including SBS modified asphalt was prepared. Star-SBS was added to the blend at 4% by weight of SBS modified asphalt binder. Blending was performed in two steps; first, SNB was mixed by shear blending at the speed of 1000 rpm at a temperature of 170 °C for 15 min; then, the second round of mixing was imposed with a high shear blending at the speed of 5000 rpm for an additional 45 min. Each batch of blend consisted of approximately 1.2 kg of SBS modified binder.

### 3.2. Experimental Program for SNB and SNB Modified Asphalt Binder

The SNB additive was first evaluated in terms of physical properties. For this purpose, the amount of ash in the SNB was obtained according to the current standard on the bitumen ash content test at 900 °C (JTG E20 T0614-2011) [41]. In addition, the actual asphalt binder content of the SNB additive was obtained by ignition at 538 °C (JTG E20 T0735-2011) [42] as conventionally performed for hot mix asphalt (HMA). The ignition method was selected because a high content of asphalt binder was expected in the SNB; this could be a source of technical issues for the centrifuge of the extractor, causing potential damage. After ignition, the gradation of the reaming particles and impurities obtained from the SNB material was conducted. The conventional characteristics of the modified SNB asphalt binder were estimated based on penetration (JTG E20 T0604-2011) [32] softening point—Ring and Ball (JTG E20 T0606-2011) [43] and ductility tests (JTG E20 T0605-2011) [44].

The softening point difference test is an experiment that can quantitatively evaluate the storage stability of asphalt (JTG E20 T0661-2011) [45]. In this method, the asphalt binder is placed into the specified test tube and positioned vertically at 163 ± 1 °C for 48 h. Then, the tube is stored in a refrigerator at −6.7 ± 5 °C for at least 4 h. Next, the specimen is exposed to room temperature and as the material temperature increases slightly, the sample is equally divided into three parts. The top and bottom one-third samples are used for the softening point test. The smaller the softening point difference (SPD) between the top sample and the bottom sample, the better the storage stability of modified asphalt, and vice versa.

The overall rheological behavior was characterized by performing frequency sweep tests with a dynamic shear rheometer (DSR). The temperature was set from 4 °C to 76 °C, while frequencies covered a range between 20 Hz to 0.1 Hz. The tests were performed with a plate–plate geometry: an 8 mm diameter was used for the lowest testing temperatures, while 25 mm plates were adopted for higher temperatures. Before testing, the binder was short-term aged according to the rolling thin-film oven test (RTFOT) procedure (JTG E20 T0610-2011) [46]. To determine the testing strain level allowing the binder response to remain within a linear viscoelasticity range, an amplitude sweep was conducted before frequency sweep tests. Then, the DSR measurements were used to generate the master curves of the shear modulus |G*| for both the original Pen 60/70, SBS modified binder, and the SNB modified binders.

Two methods were selected to address the high temperature performance of asphalt binder blends: Superpave rutting factor (G*/sinδ) (JTG E20 T0628-2011) [33] and multiple stress creep recovery (MSCR) test at T = 64 °C (AASHTO T350-2019) [34]. The DSR tests for the Superpave rutting factor were performed on both unaged and short-term aged (JTG E20 T0610-2011) [46] material. The testing frequency was set to 10 rad/s, while an initial temperature of T = 64 °C was adopted and then increased or decreased by 6 °C intervals until the value of G*/sinδ was less than or equal to 1.0 kPa for unaged binders and 2.2 kPa for RTFOT aged binders (JTG E20 T0610-2011) [46], respectively. The conventional two stress levels of 0.1 kPa and 3.2 kPa were adopted for the MSCR procedure [34]. The temperature was set to 64 °C and the test was conducted on RTFOT aged binders. During testing, the sample was subjected to twenty cycles consisting of 1 s of creep stress followed by 9 s of recovery for the 0.1 kPa stress level and ten for the 3.2 kPa stress level.

At high temperature, zero shear viscosity (ZSV) is closely related to the rutting resistance of asphalt material, which can be used to evaluate the high temperature performance. ZSV is the viscosity measured when the shear rate is close to zero, usually represented by η_0_. A dynamic shear rheometer (DSR) was used to carry out the frequency sweep test at 60 °C (referring to the temperature of rutting test); 25 mm plates were used, the thickness of the sample was kept equal to 1 mm, and the frequency range was set to 100–0.1 rad/s. Based on the simplified Cross/Sybilski model [47,48], the zero shear viscosity of the asphalt binder was obtained by fitting the frequency sweep results.

At intermediate temperature, the linear amplitude sweep (LAS) method (AASHTO TP101, 2014) [35] was selected to evaluate the fatigue behavior of the SNB modified binders. The test was performed at 25 °C with a frequency ranging between 0.2 and 30 Hz at a strain level of 0.1%, followed by a linear amplitude sweep over a range of 0.1–30% strain at a frequency of 10 Hz. The viscoelastic continuum damage (VECD) model [49] was next used to derive the number of cycles to failure at strain levels of 2.5% and 5%.

Creep tests were conducted with the bending beam rheometer (BBR) (JTG E20 T0627-2011) [36] to investigate the low temperature performance of the asphalt binder blends. Before testing, binders were long-term aged with a pressure aging vessel (PAV) (JTG E20 T0630-2011) [50]. Measurements were performed starting from an initial temperature T = −18 °C and next by varying it in steps of 6 °C until the conditions (creep stiffness, S (t = 60 s) ≤ 300 MPa and *m*-value ≥ 0.300) were met. If the deformation of the specimen was more than 4 mm or less than 0.08 mm, the experimental results were not recorded.

The response of the SNB modified binder at mixing temperature was evaluated with a rotational viscometer and the dynamic viscosity (JTG E20 T0625-2011) [37] at 135 °C and 165 °C was recorded for the different binder blends.

FM, FTIR, and GPC tests were performed on virgin and SNB modified asphalt to investigate the modification mechanism of the SNB modifier on asphalts.

The distribution of the SNB modifier can be studied by fluorescence microscopy (FM). When the light excited by the fluorescent light source is applied to the modified asphalt, the modifier will reflect light under the excitation of fluorescence, while the asphalt will not excite any light, so the modifier and asphalt can be clearly distinguished under the fluorescence microscope. The SNB modification effect and performance were evaluated by observing the shape, size, distribution uniformity of modifier, and the connection with asphalt.

The infrared spectrum of the virgin asphalt and SNB modified asphalt was obtained by detecting the absorption of infrared rays. Fourier-transform infrared spectroscopy (FTIR) was used to obtain the chemical bonds and functional groups of SNB modifier and SNB modified binder. The sample was compressed into pellets with a thickness of about 1 mm and then placed in the instrument. Through scanning by FTIR spectrometer, the infrared spectrum range of 4000 to 400 cm^−1^ of the sample was obtained.

The molecular weight distribution of SNB modified asphalt was obtained by a gel permeation chromatography (GPC) test. GPC is a liquid chromatography method that uses the unique characteristics of porous gel stationary phases to separate molecules of different sizes according to the elution time. This study used tetrahydrofuran as the mobile phase; the temperatures of the columns were 35 °C, the flow rate was 1.0 mL/min, the concentration of the test solution was 2.0 mg/mL, and the injection volume was 10 μL. Table 2 summarizes the experimental program on asphalt binder for the present research. All the experiments mentioned above were carried out on three replicates.

### 3.3. Physical Properties

The results of the Mineral Matter or Ash in Asphalt Materials assay (JTG E20 T0614-2011) [41] demonstrated that the ash content of SNB is 15.41% at 900 °C. Based on the ignition process (JTG E20 T0735-2011) [42], at 538 °C, a particle content equal to 19.50% was observed for the SNB while the asphalt content was found to be 80.50%. The particles and impurities obtained after the ignition process were subjected to gradation analysis (JTG E20 T0327-2011) and the results are reported in Table 3, suggesting the presence of fine material in the SNB with a size range between 0.3 mm and the filler.

### 3.4. Conventional Properties

**Figure 3:**
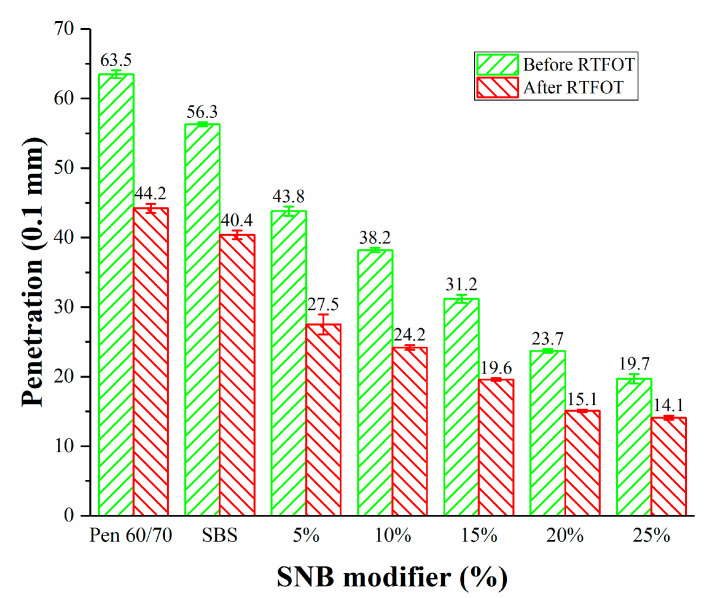
Penetration at 25 °C for the entire set of binder blends (JTG E20 T0604-2011).

**Figure 4:**
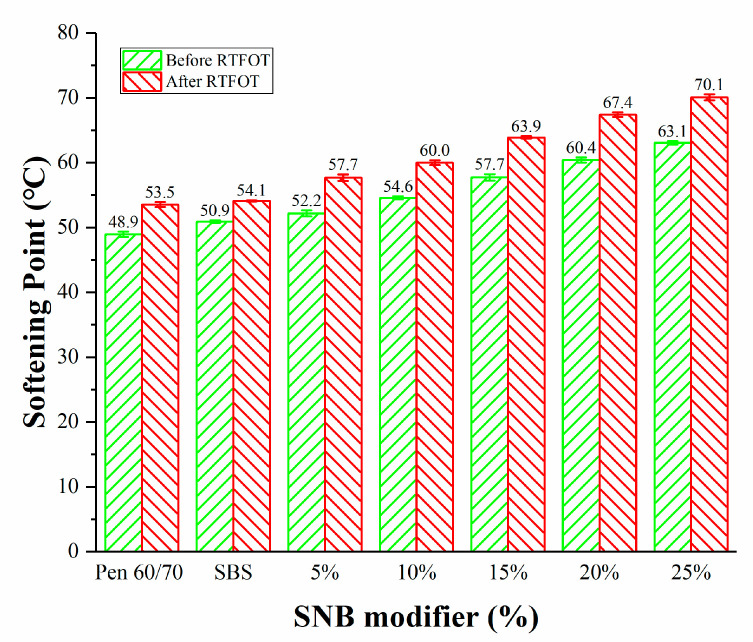
Softening point for the entire set of binder blends (JTG E20 T0606-2011).

**Figure 5:**
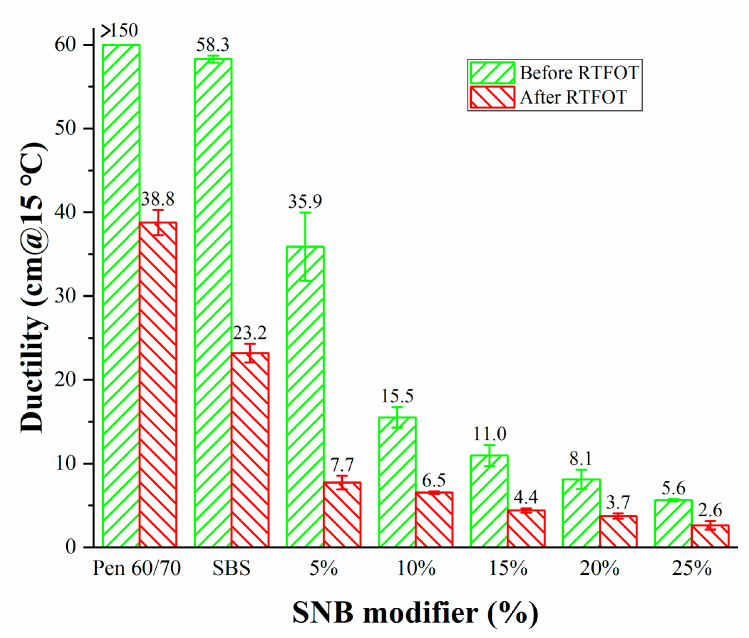
Ductility for the entire set of binder blends (JTG E20 T0605-2011).

The results of the penetration (25 °C) (JTG E20 T0604-2011) [32], softening point (JTG E20 T 0606-2011) [43], and ductility (T=15 °C) (JTG E20 T 0605-2011) [44] tests are shown in Figure 3, Figure 4 and Figure 5. The addition of SNB led to lower penetration and a higher softening point. This evolution was confirmed by the measurements obtained from the ductility test which showed values below 40 cm for SNB modified material. This is considerably lower than what was observed in the case of the Pen 60/70 binder with ductility greater than 150 cm and the SBS modified binder with ductility equal to 58.3 cm. A similar trend can be observed in the case of the short-term aged samples, for which the aging resulted in harder and less ductile material compared to the unaged condition. This suggests that the presence of the SNB additive may potentially negatively affect the low-temperature performance of the binder blends both in the unaged and short-term aged conditions. The experimental results of the conventional tests support the idea that SNB can decrease the penetration, increase the softening point, and decrease the ductility of asphalt. This is consistent with the results of BRA modified asphalt [21,22,23,24].

**Figure 6:**
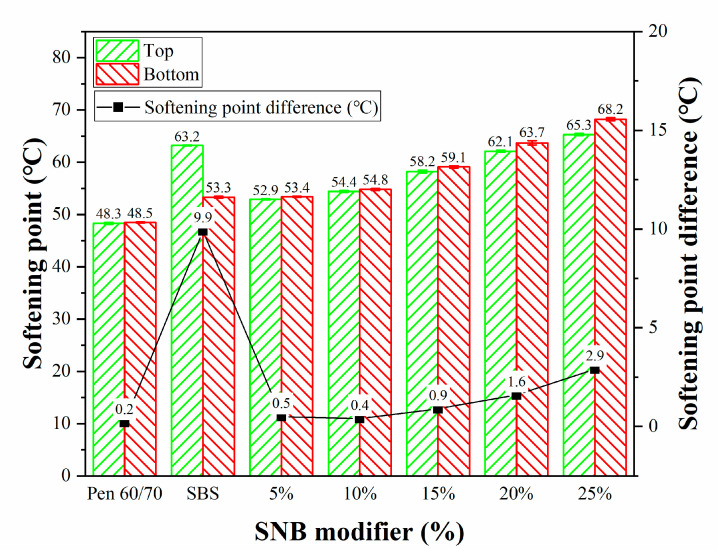
Softening point difference for the entire set of binder blends (JTG E20 T0661-2011).

It can be seen from Figure 6 that the softening point difference (SPD) of base asphalt is the lowest, while that of SBS modified asphalt is the largest, which indicates that the storage stability of SBS modified asphalt is the worst, and a stabilizer needs to be added. The SPD of 5% and 10% is smaller and close to that of the base asphalt, which means that the smaller SNB content has less influence on the storage stability of asphalt. It can also be noted that the SPD and the difference of SPD of modified asphalt with SNB content more than 10% increases with the increase in SNB content, which indicates that higher SNB content is unfavorable to the storage stability of asphalt. According to the existing Chinese standard JTG F40-2004 [51], the softening point difference of modified asphalt should not be more than 2.5 °C; however, the SPD of 25 wt.% SNB modified asphalt was 2.9 °C, which did not meet the requirement from the standard. Therefore, it is not recommended for use.

### 3.5. Rheological Properties

#### 3.5.1. Overall Rheological Behavior

**Figure 7:**
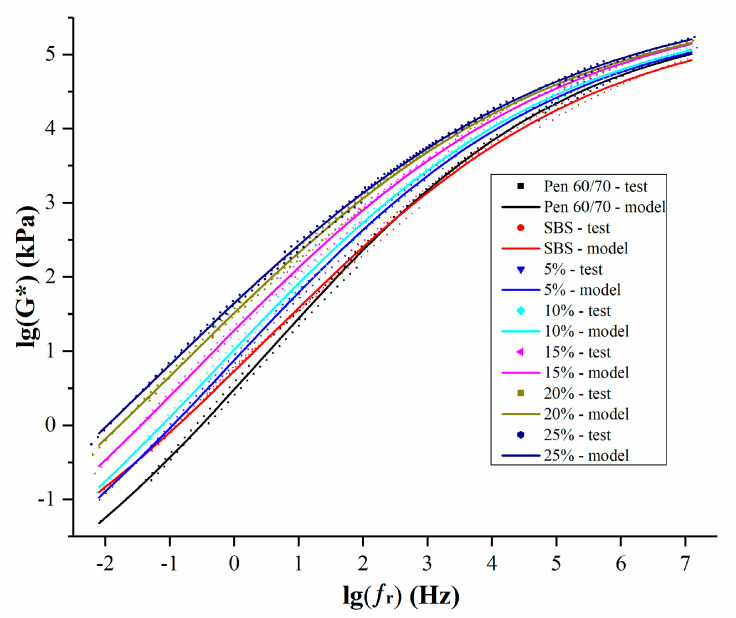
Master curve of tested asphalt binders.

The measurements obtained from the temperature–frequency sweep tests performed on the SNB modified binders were used to generate the master curve of the complex shear modulus (G*) based on the time–temperature superposition principle (TTSP) [52]. For this purpose, a reference temperature of 60 °C was selected. This is the same temperature also used during the rutting tests on asphalt mixtures (JTG E20 T0719-2011) [40]. The frequency sweep test data were fitted with a simple sigmoidal function (Equation (1)) [52] to obtain the master curve of G*:

(1) $$log|G*(f)|=\delta +\frac{\alpha }{1+e^{\beta +\gamma \cdot Logf_{r}}}$$

where *f* is the test frequency at specific temperatures, *δ* is the minimum modulus value, *α* is the span of G*, *β* and *γ* are the shape parameters, and *f_r_* is the reduced frequency at reference temperatures (60 °C). The reduced frequency was obtained from the actual frequency based on Equation (2) and relying on a shift factor (*a(T)*) that can be derived from the well-known Williams–Landel–Ferry (WLF) formula (Equation (3)) [53].

(2) $$log(f_{r})=log(f)+log(a(T))$$

(3) $$log(a(T))=\frac{-C_{1}\cdot \Delta T}{C_{2}+\Delta T}$$

where *C_1_* and *C_2_* are model constants, and ∆*T* is the difference between the test temperature and reference temperature. The master curves of G* of the unmodified Pen 60/70, SBS and SNB modified binder are shown in Figure 7, while the model parameters are presented in Table 4.

Higher frequency resulted in increased complex shear modulus. According to the time–temperature superposition principle (TTSP), the impact of low frequency on the pavement is equal to the impact of high temperature on the pavement, and vice versa. Compared with base asphalt, SNB modified binders showed higher moduli in almost all frequency ranges, suggesting better rutting resistance but poorer behavior against low temperature cracking. Compared with SBS modified asphalt, the low temperature performance of SNB modified asphalt was worse in the high frequency domain, while it showed better high temperature performance at low frequency when the SNB content was more than 5%. This phenomenon is more remarkable for a higher amount of SNB modifier. A more detailed evaluation of the material response at high, intermediate, and low temperatures is presented in the next three sections.

#### 3.5.2. High Temperature Performance

**Figure 8:**
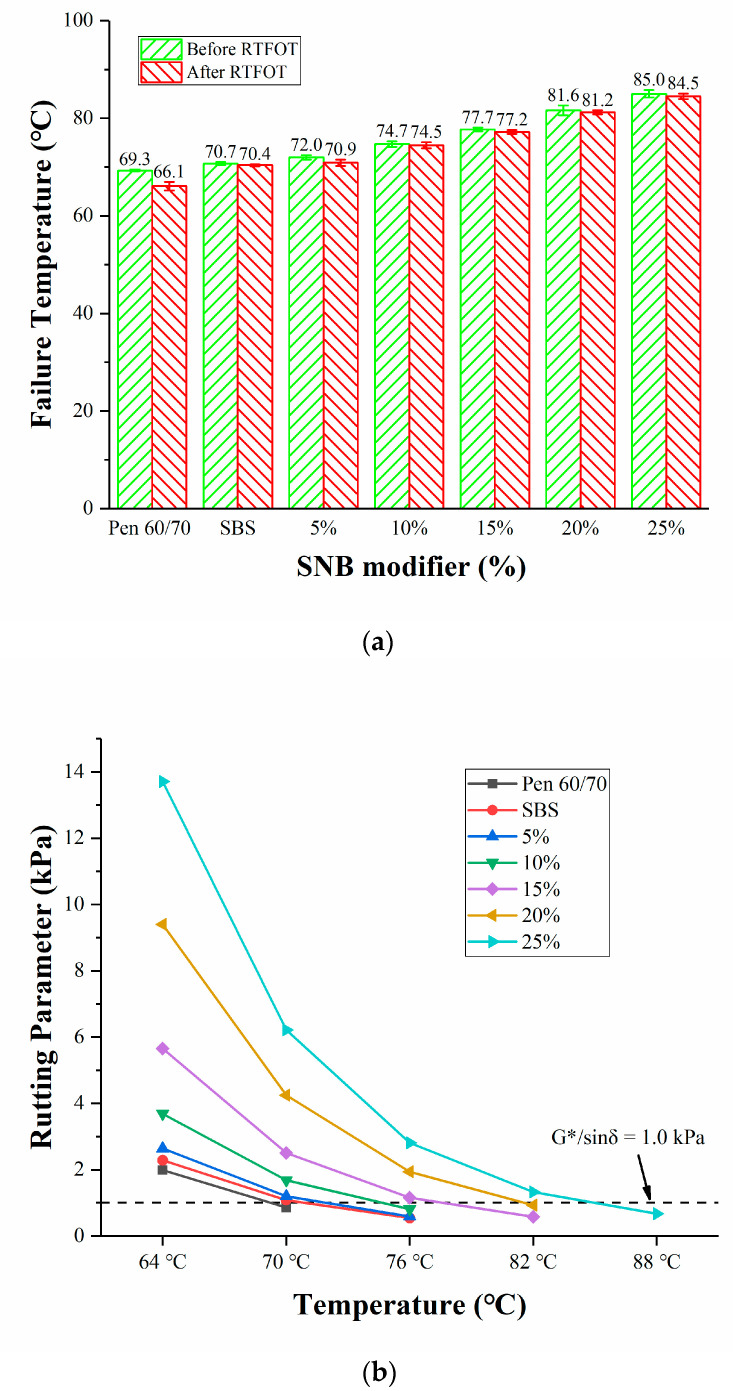

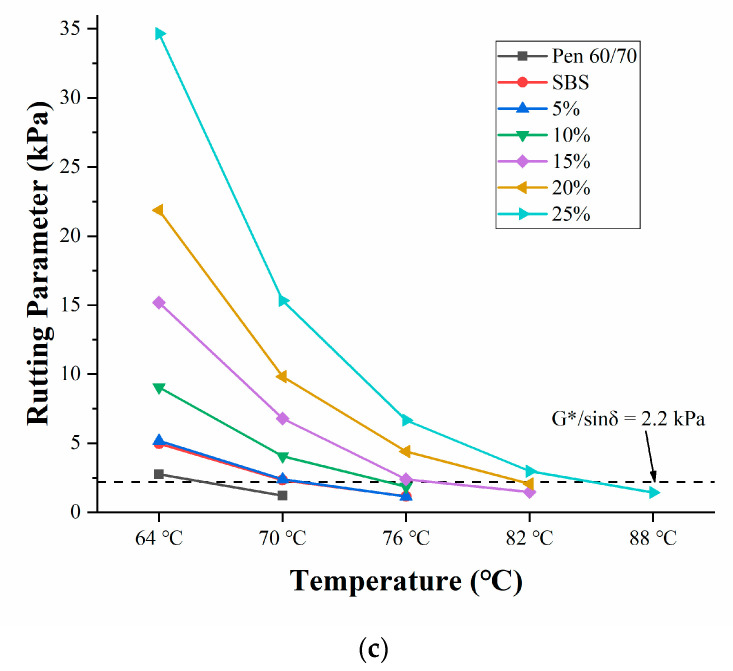
Results of Superpave rutting factor test: (**a**) failure temperature; (**b**) Superpave rutting factors for unaged samples; (**c**) Superpave rutting factors for RTFOT samples.

The high temperature behavior of the set of SNB modified asphalt binders was evaluated both with the Superpave rutting factor (JTG E20 T0628-2011) [33] and through the MSCR test (AASHTO T350-2019) [34]. Figure 8 presents the failure temperatures together with the rutting factors at each testing temperature. It can be observed that failure temperatures of RTFOT aged SNB binders are 0.5 to 1 °C lower than what was experienced in the case of unaged binders (Figure 8a). The largest G*/sinδ values were exhibited by the binder blend with the highest content of SNB (25 wt.%), which indicates potentially better performance against rutting phenomena (Figure 8b,c). Compared to Pen 60/70 and SBS asphalt, all SNB modified blends exhibited a considerably better response at high temperature. Consistently with the softening point measurements, the failure temperature increased for higher SNB content.

To further evaluate the rutting response of the binder blends, MSCR tests were performed according to the current standard (AASHTO T350-2019) [34]. Average percent recovery, R, non-recoverable creep compliance, J_nr_, and stress sensitivity, J_nr-diff_, were computed and used for the analysis. The percent recovery provides information on the elastic response of the material, where higher values of R are observed for a more elastic-like behavior. Superior capability to resist permanent deformation is associated to lower J_nr_, while the stress sensitivity of asphalt binder can be evaluated through parameter J_nr-diff_. Except for SBS modified asphalt, a substantial agreement between the Superpave factor G*/sinδ and the results of the MSCR tests was shown by the values reported in Table 5. It can be seen from Table 5 that the J_nr_ value of SNB modified asphalt with more than 5% content is smaller, which means that they have better high temperature performance than SBS modified asphalt. Superior response to permanent deformation associated with a more remarkable elastic behavior can be observed for the SNB modified material. Specifically, the original binder and the binder modified with 5 wt.% and 10 wt.% of SNB were capable of meeting the requirements for the Heavy Traffic “H” grade according to the AASHTO M332-2019 [54]. For the intermediate degree of modification (15 wt.% of SNB), the Very High Traffic “V” grade could be satisfied. When the amount of SNB in the range of 20–25 wt.% was incorporated in the blend, the modified binder could meet the performance criteria for the Extremely Heavy Traffic “E” grade.

Anderson et al. (2001) found that the performance of modified asphalt at high temperature can not be well evaluated by SHRP rutting factor G*/sinδ [55]. Sybilski et al. (1993) found that for asphalt, the viscoelastic test results are closely related to the shear rate, and the best result to reflect the material’s characteristics is the case of zero shear rate in the ideal state [56]. Therefore, the concept of zero shear viscosity (ZSV) in non-Newtonian fluid theory was introduced into polymer modified asphalt to evaluate the high temperature performance of asphalt binder [56]. The frequency sweep test data were fitted with a simplified Cross/Sybilski formula [47,48], as shown in Equation (4), to obtain the ZSV.

(4) $$\eta^{*}=\frac{\eta_{0}}{1+{\left(K\omega \right)}^{m}}$$

where η* is the complex shear viscosity (Pa.s), *η*_0_ is the zero shear viscosity (Pa.s), *w* is the frequency (rad/s), and *K* and *m* are the fitting coefficients related to the material.

**Figure 9:**
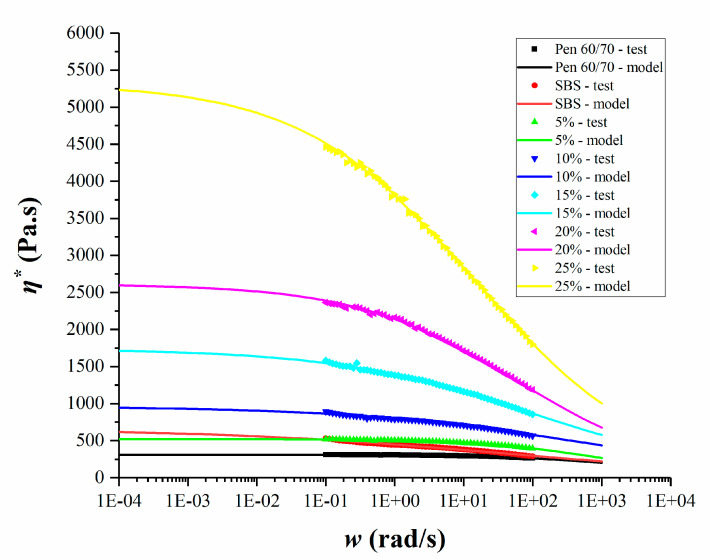
Viscosity curve of tested asphalt binders.

The model parameters are presented in Table 6. It can be seen from Figure 9 that the zero-shear viscosity increases with the increase in the content of SNB. Only when the SNB content is more than 5 wt.%, is the zero-shear viscosity of all modified asphalts higher than that of SBS modified asphalt. These results are consistent with those of the MSCR test, and were anticipated with the rutting test of the mixture (see Section 4), rather than the rutting factor test results of the binder. This further proves that the points of view of Anderson and Sybilski were correct on the limitations of the rutting factor test of asphalt mixture [55,56].

It was also noted that, with the increase in SNB content, the difference of zero shear viscosity of SNB modified asphalt became larger. For example, the zero-shear viscosity of 25 wt.% SNB modified asphalt was more than two-fold greater than that of 20 wt.% SNB modified asphalt. This means that ZSV test can distinguish the high temperature performance of modified asphalt with a high content of SNB. The experimental results of the three high temperature performance tests conducted above show that SNB can improve the high temperature performance of asphalt, which is in agreement with those of other natural modified asphalt results [17,18,21].

#### 3.5.3. Fatigue Performance

**Figure 10:**
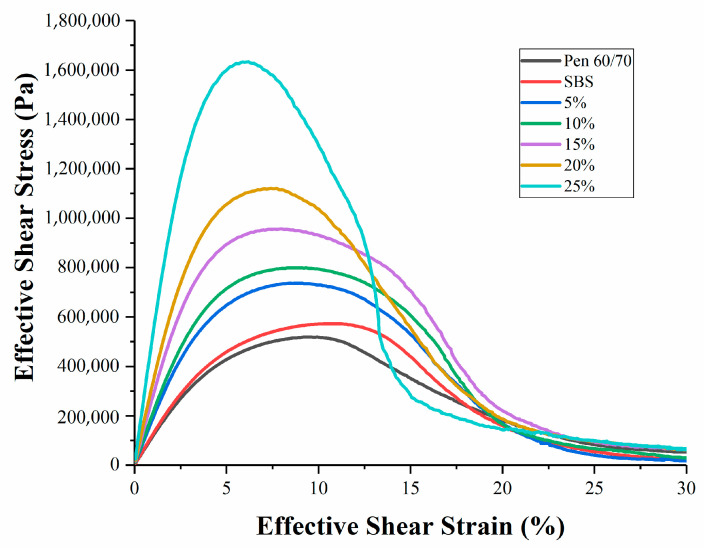
Stress–strain curve of the linear amplitude sweep (LAS) test.

**Figure 11:**
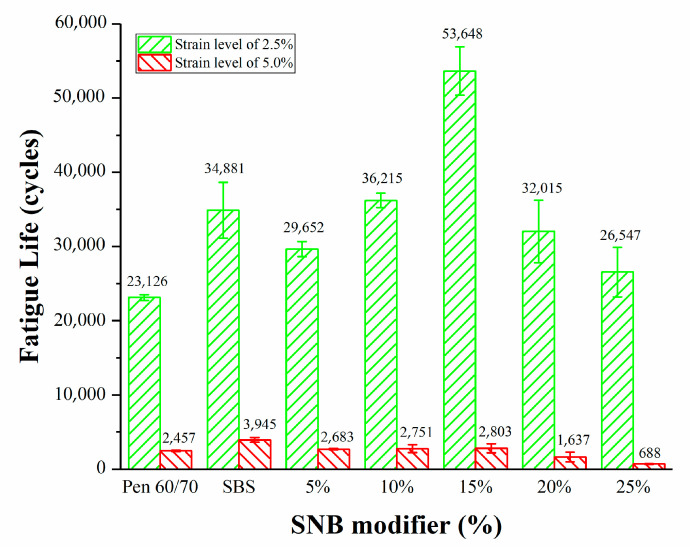
Linear amplitude sweep (LAS) test results.

There are very few studies about the fatigue performance of natural modified asphalt. The LAS test was selected for addressing the fatigue performance of the SNB modified binders at a testing temperature of 25 °C (AASHTO TP101) [35]. According to the requirements of AASHTO TP101 (2014) [35], the value corresponding to the peak point of the stress–strain curve is regarded as the fatigue failure point. The stress–strain curves of blends are shown in Figure 10. It can be seen from the curves that the strain corresponding to the fatigue failure point decreases with the increase in SNB content. Under the same strain condition, the stress increases with the increase in SNB content before reaching the fatigue failure point. The fatigue life, represented by the number of loading cycles to failure (Nf) at the strain levels of 2.5% and 5.0%, is presented in Table 7 and Figure 11.

For the specific strain level, higher values of Nf were associated with better fatigue life. The binder blend containing 15 wt.% of SNB exhibited the best fatigue performance, with an improvement of 132% and 14% in terms Nf compared to the original Pen 60/70 binder for the strain levels of 2.5% and 5%, respectively. Moreover, the binder blend containing 15 wt.% of SNB showed an improvement of 54% in terms of Nf compared to the original SBS binder for the strain level of 2.5%. However, at a 2.5% strain level, the fatigue life of 5 wt.%, 10 wt.% and 25 wt.% blends were lower than that observed for the SBS modified asphalts, whereas at the 5% strain level, the fatigue lives of all blends were lower than that of SBS modified asphalts. This means that, in general, the fatigue life of SNB modified asphalt is not as long as that of SBS modified asphalt.

At the two strain levels, the fatigue life first increases and then decreases with the increase in SNB. Overall, the fatigue resistance increased up to an optimal content of SNB of 15 wt.%; beyond this threshold (i.e., for 20 wt.% SNB and 25 wt.% SNB) a decrease in Nf was experienced. One possible explanation for the decline in fatigue life may be associated with the increased amount of impurity in the binder blend, which deteriorated the fatigue performance due to higher material stiffness and potentially caused an increase in brittleness.

#### 3.5.4. Low Temperature Performance

The low temperature response of the binder blends was evaluated by assessing creep stiffness S (t = 60 s) and corresponding relaxation parameter *m*-value obtained from the bending beam rheometer (BBR) test (JTG E20 T0627-2011) [36]. The low performance grade (PG) can then be determined based on the combined criteria for which S (t = 60 s) < 300 MPa, while the *m*-value > 0.3. Higher stiffness values are expected to result in higher susceptibility to low-temperature cracking. The values of S (t = 60 s) and the *m*-value obtained at T = −18 °C, −12 °C, −6 °C, and 0 °C are reported in Table 8.

Based on the results of the tests, a negative impact of the SNB modifier can be observed on the low temperature performance. This due to an increase in creep stiffness and a corresponding decrease in the *m*-value. This implies that the binder blend presents reduced relaxation capabilities which may be associated with potentially higher brittleness. Such a trend is exemplified by the increase in the low PG for the different degrees of modification: low PG = −22 °C for virgin binder; low PG = −22 °C for SBS binder; low PG = −16 °C for SNB content between 5 and 20 wt.%; and low PG = −10 °C when 25 wt.% of SNB was incorporated in the virgin binder.

It must be remarked that the presence of impurities and small particles in the SNB materials makes the blend of Pen60/70 binder and SNB a peculiar material that cannot be necessarily identified as a binder but rather as a mastic. Therefore, the increase in stiffness and the corresponding decrease in *m*-value may be partially associated with this characteristic.

#### 3.5.5. Workability

**Figure 12:**
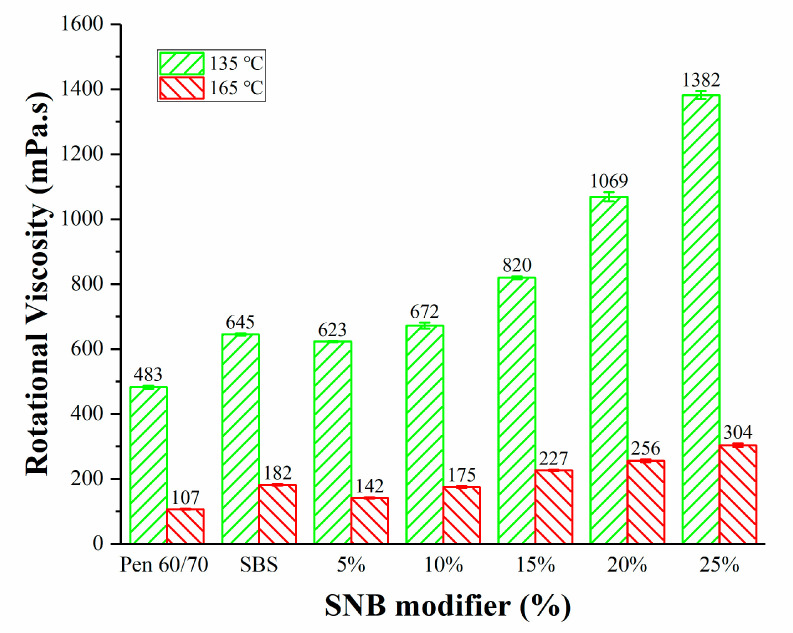
Rotational viscosity for all sets of binder blends (JTG E20 T0625-2011).

A critical aspect of asphalt mixture is related to its workability. This term can be used to describe several properties linked to different phases of pavement construction, such as production, delivery, laying, and compaction. Better workability is also associated with costs and sustainability, because the movement and handling of the mixture may require less energy overall, with obvious economic and environmental benefits. To indirectly evaluate the workability of the material at the binder level, the rotational viscosity of the different binder blends was measured, and the results are reported in Figure 12. The presence of the SNB modifier induced an increase in the rotational viscosity both at 135 °C and 165 °C with larger values for higher SNB content.

According to the current standard of JTG E20 T0625 -2011 [37] in China, asphalt mixing viscosity is limited to 170 ± 20 mPa.s. For this reason, the selection of the SNB content to be used in asphalt mixture has to be taken into consideration, not only in terms of performance-related parameters but also in terms of material workability. Following the current standard (JTG E20 T0625-2011) [37], the mixing temperature (Table 9) was adjusted to meet this criterion for the different SNB contents for the preparation of the SMA-10 mixture, which is present later in this manuscript.

### 3.6. Mechanism Investigation

#### 3.6.1. Morphology

**Figure 13:**
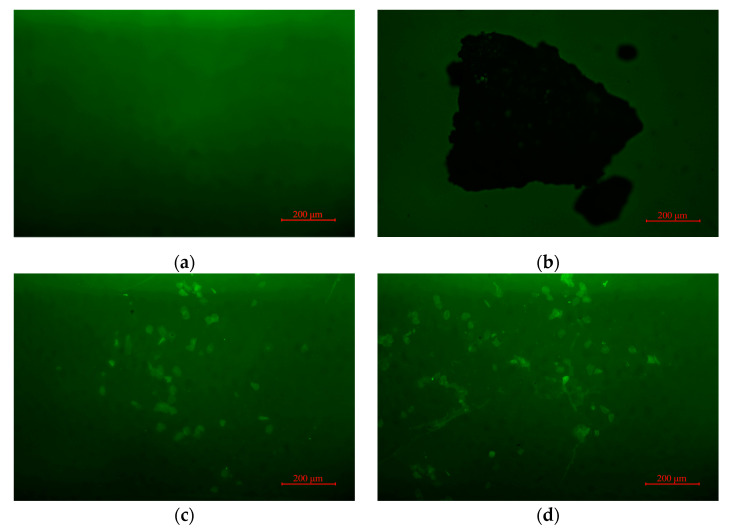
The FM images: (**a**) Pen 60/70; (**b**) SNB modifier; (**c**) 10 wt.% SNB modified asphalt; and (**d**) 20 wt.% SNB modified asphalt.

Figure 13a shows the FM image of the base asphalt. Figure 13b shows the morphology of SNB modifier. The black part is the asphalt part of SNB, and the light spot of SNB is the silicon dioxide and other impurities of SNB. In the natural state, asphalt is coated with silica and other impurities, which together constitute the SNB modifier. In the process of preparing SNB modified asphalt, the SNB modifier in Figure 13b is heated, part of the asphalt is heated and melted into the base asphalt, while impurities such as silica are not melted. Finally, light spots in Figure 13c,d are shown. It can be seen from Figure 13c,d that the distribution of the SNB modifier after dissolving in SNB modified asphalt is uneven, and the uneven distribution becomes more obvious with the increase in SNB content. The distribution of the modifier after melting is granular, agglomerate, and flocculent. Part of the SNB modifier dissolved to form granular morphology. In 10 wt.% and 20 wt.% SNB modified asphalt, there are agglomerate and flocculent morphologies, which is because the asphalt with a larger molecular weight in SNB is not easy to dissolve when heated, and adheres to impurities such as silica, so their adhesion force is stronger, which makes impurities such as silica aggregate into agglomerate and flocculent morphology. This indicated that after dissolving, the size of the SNB modifier becomes smaller and the distribution of SNB modifier is uneven, showing three forms, granular, agglomerate and flocculent properties.

#### 3.6.2. Chemical Properties

**Figure 14:**
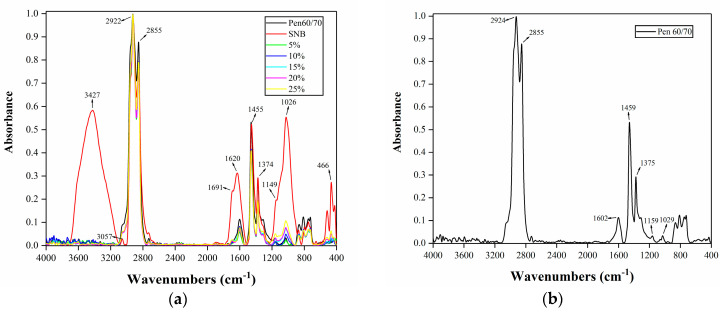

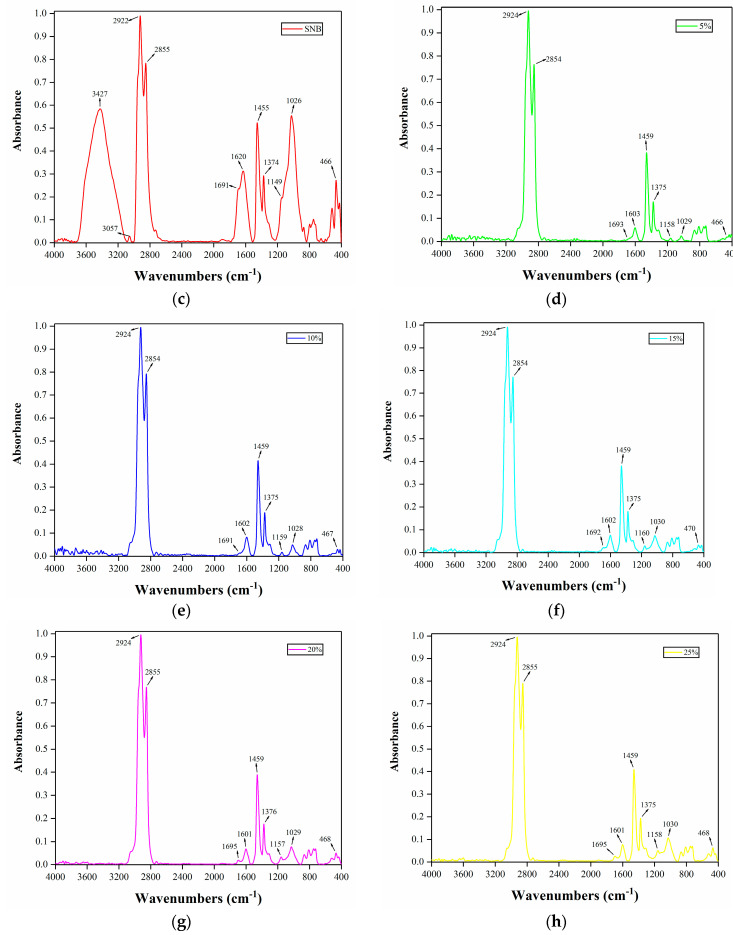
Result of FTIR tests: (**a**) all binder; (**b**) Pen 60/70; (**c**) SNB; (**d**) 5% SNB; (**e**) 10% SNB; (**f**) 15% SNB; (**g**) 20% SNB; and (**h**) 25% SNB.

The smoothed and normalized infrared spectrum of the binders are shown in Figure 14. According to Figure 14b,c, the peak shape of the SNB modifier is generally the same as that of Pen 60/70. This means that their chemical components are very similar. Typically, the peak at 3427 cm^−1^ is the O–H stretching vibration of water molecules, which does not exist in other asphalts. This means that SNB contains moisture in its natural state. The moisture in the SNB modifier should be removed both for scientific research and production purposes to prevent the potential negative effect on the material performance. Figure 14c shows a peak at 3057 cm^−1^, which is the =CH stretching vibration of the benzene ring. This peak is obvious in SNB, but not in SNB modified asphalt. A possible reason is that during the stirring process, a chemical reaction occurs to generate other functional groups.

The bands at 2922–2924 cm^−1^ correspond to the asymmetric stretching vibration of C–H in saturated hydrocarbon, and the range at 2854–2855 cm^−1^ corresponds to the symmetric stretching vibration of C–H in saturated hydrocarbon. Peaks at 1455–1459 cm^−1^ are the deformation vibration of C–H, which are the deformation of methylenes (–CH_2_–) and the asymmetric deformation of the methyl group (–CH_3_). Peaks at 1374–1375 cm^−1^ are the deformation vibration of C–H, which is the symmetric deformation of the methyl group (–CH_3_). These are the most typical peaks of asphalt. Peaks at 1601–1620 cm^−1^ are the skeleton vibration of C=C in substituted benzene. The values of peak area and height are very high in the SNB modifier, whereas the values are different in other SNB modified asphalt. This means that the addition of SNB has a greater impact on the structural components of aromatic hydrocarbons such as benzene rings.

Peaks at 1691–1695 cm^−1^ are the stretching vibration of the carbonyl group (C=O). High values of peak area and height in SNB modifier shows that SNB modifier has a high degree of aging in its natural state. Moreover, the SNB modifier contains a large number of oxygen-containing groups and polar functional groups such as aldehydes, ketones, esters, and carboxylic acids, among others. The peak area and height of 5 wt.% and 10 wt.% SNB modified asphalt are very low, indicating that their SNB contents are less. It can also be noticed that these peaks do not exist in the virgin asphalt. The range 1149–1160 cm^−1^ corresponds to the stretching vibration peaks of C–O. This will oxidize when the asphalt is aged and finally change into C=O (1691–1695 cm^−1^). It can be observed from Figure 12 that the higher the SNB dosages, the higher the value of peak area and height. This means that the aging degree of SNB modified asphalt is higher after adding SNB. Peaks at 1026–1030 cm^−1^ are the stretching vibration of the sulfoxide group (S=O). Figure 12 reveals that the higher the SNB dosages, the higher the value of peak area and height. The reason for the formation of the sulfoxide group (S=O) is that the sulfur-containing functional group in asphalt reacts violently with oxygen during aging.

The ability of SNB modified asphalts to resist external forces has been improved, which might be because of two reasons. Firstly, the oxygen atoms of the sulfoxide group (S=O) and the carbonyl (C=O) can easily form hydrogen bonds with the hydrogen atoms in other functional groups, which makes the asphalt molecules more tightly bound. Secondly, the sulfoxide group (S=O) and the carbonyl (C=O) are polar functional groups, with a permanent dipole having electrostatic force between the polar dipoles, promoting the bonding between molecules and increasing the Van der Waals force. Existing studies show that the sulfur group (S=O) and the carbon (C=O) are positively correlated to the viscosity, softening point, and rheological properties of asphalt [57,58,59]. Specifically, with the increase in SNB content, the softening point, rutting factor, and viscosity of SNB modified asphalt also increase.

The peaks from 466 cm^−1^ to 470 cm^−1^ are the symmetric stretching vibration of Si–O. SNB has a high silica content, while virgin asphalt does not. In the previous physical properties test (JTG E20 T0735-2011) [42], the remaining impurity of SNB after the ignition was silica. Figure 14 depicts that the higher the amount of SNB, the higher the value of peak area and height will be. When preparing SNB modified asphalt, the asphalt components in SNB will dissolve in pure asphalt, but silica will not dissolve, acting as an impurity, resulting in a reduction in ductility by causing stress concentration and making specimens bear uneven force.

#### 3.6.3. Molecular Composition

**Figure 15:**
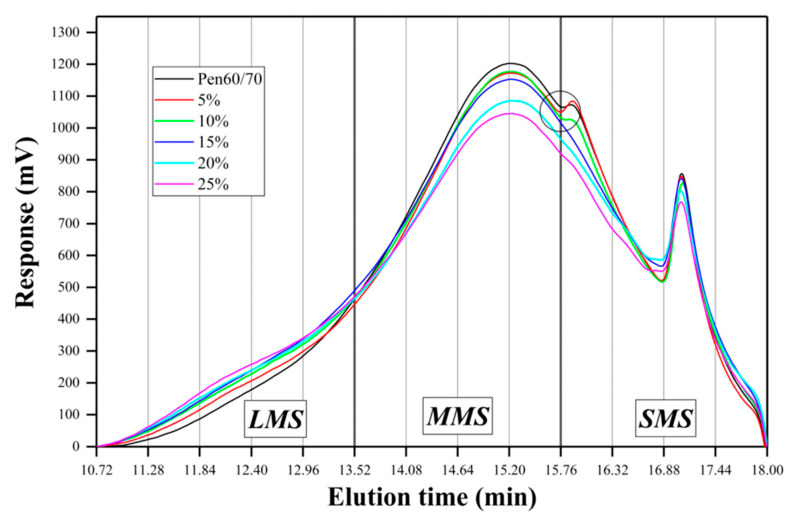
Result of the GPC test.

Figure 15 shows the GPC test results of virgin and SNB modified asphalt binders. In the GPC experiment, the components with large molecular weight travel in the chromatographic column with a shorter distance and shorter elution time, and vice versa. Therefore, by analyzing the fluctuation of the GPC test curve, the relative content of a specific molecular weight component can be obtained.

Jennings et al. (1980,1985) [60,61] and Kim et al. (2006) [62] proposed a method to analyze GPC experiments, which is the cutting method of the area enclosed by the elution curve and the baseline. After the baseline is determined in the spectrum, the area enclosed by the baseline and the elution curve is cut equidistantly according to the elution time. According to Kim’s study (2006) [62], the entire area of 13 slices of each sample was adjusted to 100%, and then the molecular weight distribution was compared using the area ratio. As Figure 15 shows, the elution curve from 10.72 to 18 min (referring to the molecular weight range from 22813 to 116 g/mol) is equally divided into 13 pieces. A larger percentage of specific molecular size is represented by a higher area ratio. There are 1–5 slices defined as large molecule size (LMS), 6–9 slices as middle molecule size (MMS), and 10–13 slices as small molecule size (SMS). According to the sum of the area of different slices, the ratio of LMS, MMS, and SMS can be calculated. It can be seen from Table 10 that with the increase in SNB content, the proportion of LMS gradually increases, while the proportion of MMS gradually decreases, and the proportion of SMS changes little. This is also the modification mechanism of SNB modified asphalt.

The relative amount of LMS in the GPC test is closely associated with the absolute viscosity, kinematics viscosity, and penetration, as stated in many research studies [62,63,64]. As Table 10 shows, with the increase in SNB content, the ratio of LMS keeps increasing, leading to an increase in softening point and rotational viscosity, while the penetration decreases.

It can be observed from Figure 15 that at 15.76–16.32 min, there is a small peak at Pen, 5 wt.%, and 10 wt.%. This peak is one of the small molecule size components. With the increase in SNB content, this peak (SMS components) disappeared, which might be because SNB has changed the composition of asphalt, and the SMS components of this part have been transformed into the components of the other parts. According to Kim’s approach (2006) [62], at 15.76 min, the dividing line just passed the lowest point of the two peaks of MMS and SMS for Pen 60/70, 5 wt.% and 10 wt.%, which also means that Kim’s method is suitable for virgin and SNB modified asphalts.

Table 11 displays the results of GPC experiments based on statistical analysis. Five parameters were selected to illustrate the molecular weight distribution of the modified asphalts, including peak molecular weight (Mp), number average molecular weight (Mn), weight average molecular weight (Mw), Z-average molecular weight (Mz), and polydispersity index (PDI = Mw/Mn). Generally, the asphalt with a wide molecular weight distribution had lower temperature sensitivity, which meant that the performance of asphalt was less affected by temperature change [65]. It can be seen from Table 11 that as the content of SNB increased, the PDI value becomes larger. This indicates that the molecular weight distribution of SNB modified asphalt became wider and less concentrated, resulting in lower temperature sensitivity of SNB modified asphalt. As the content of SNB increased, the value of Mw became larger, which means that SNB increases the proportion of high molecular weight components. In addition, the values of Mn were similar, indicating that SNB does not have a significant influence on the average molecular weight by the components with the largest number of molecules.

## 4. Investigation at the Asphalt Mixture Level

The possibility of using SNB additive was further investigated by extending this study to asphalt mixture. Given the exploratory nature of this second part of the present research, only a single asphalt mixture type was selected.

### 4.1. Preparation of Asphalt Mixture

**Figure 16:**
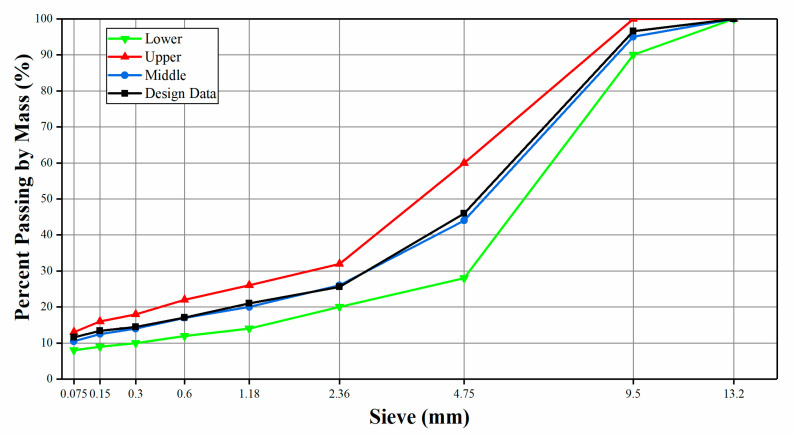
The design gradation curve of the aggregates.

In this study, a 10 mm stone mastic asphalt, SMA-10, which is commonly used in southern China (JTG F40-2004) [51], was selected as the reference material for further analysis. Table 12 presents the design gradation of the aggregate, which consisted of diabase, fine SNB material, while the filler was provided by a local asphalt plant. The filler was a limestone mineral powder, with an apparent specific gravity of 2.7. The design gradation of the aggregates is shown in Figure 16.

After the washing and gradation process, the aggregates were conditioned in an oven for 4 h at 10 °C above the mixing temperature to remove any moisture. In order to avoid the influence of different asphalt--aggregate ratios, the optimum asphalt–aggregate ratio was not adopted. By using the same asphalt–aggregate ratio, the influence of different contents of SNB modified asphalt on mixture performance could be better evaluated. The asphalt–aggregate ratio of 6.5% was adopted for virgin, SBS, and SNB modified mixtures. All five dosages of SNB evaluated during the binder experimentation phase were considered for preparing the SMA material. Mixing temperatures are listed in Table 9 in the previous section. The incorporation of the SNB modifiers was accomplished according to a wet procedure, where the asphalt binder and the SNB are first blended and then mixed with the aggregate. Compaction was performed with a Marshall hammer (JTG E20 T0709-2011) [38]; both sides of the specimen were subjected to 75 blows according to the procedure adopted in China. Together with cylindrical specimens, asphalt mixture slabs with size 300 mm × 300 mm × 50 mm were also prepared for evaluating the resistance against rutting through a wheel compaction apparatus. Control and SNB modified mixtures were finally subjected to the Marshall test (JTG E20 T0709-2011) [38], indirect tensile strength (ITS) test (JTG E20 T0729-2011) [39], and rutting tests (JTG E20 T0719-2011) [40]. Table 13 summarizes the experimental program on asphalt mixture for the present research.

### 4.2. Testing Program for Asphalt Mixture

At present, there is a lack of research on SNB using Chinese standards. Therefore, three asphalt mixture tests were conducted, including Marshall stability and flow (JTG E20 T0709-2011) [38] and indirect tensile strength (ITS) tests (JTG E20 T0729-2011) [39], to determine the moisture susceptibility and rutting resistance (JTG E20 T0719-2011) [40].

**Figure 17:**
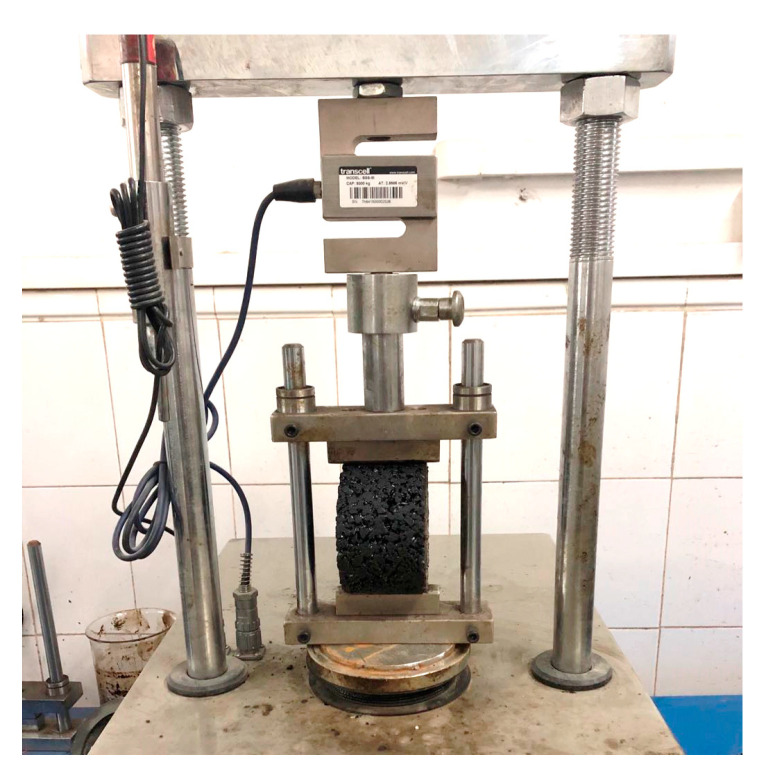
Loading frame used for the indirect tensile strength (ITS) test (JTG E20 T0729-2011).

To initially characterize the designed asphalt mixtures, Marshall stability and flow value were obtained according to the current standards (JTG E20 T0709-2011) [38]. Then, the effect of moisture susceptibility was evaluated with the ITS method by determining the ratio derived from the measurements obtained on specimens subjected to a freeze–thaw cycle and tested in their original condition (control group). A total of eight replicates per asphalt mixture were prepared: four were assigned to the control group and tested at 25 °C, while the remaining four were exposed to a “freeze” phase at −18 °C for 16 h followed by thawing at 60 °C for 24 h, before conducting the tests at the same temperature (25 °C). Before testing, all specimens underwent a conditioning phase of 2 h at the selected testing temperature, T = 25 °C in a water bath. The load was applied diametrically with a rate of 50 mm/min until the samples failed. Figure 17 presents the loading frame for the ITS test.

**Figure 18:**
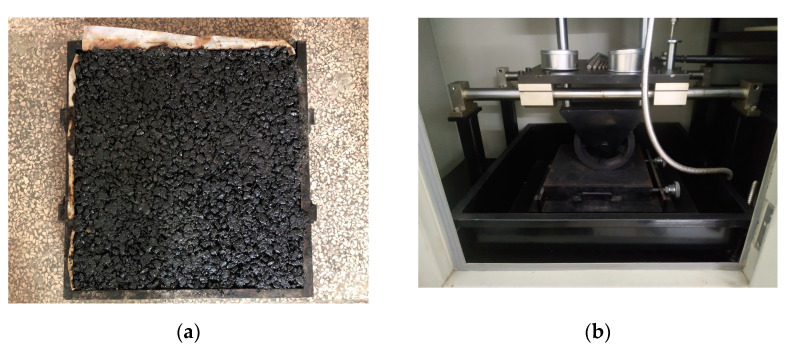
Rutting test: (**a**) specimen slab and (**b**) rutting testing device (JTG E20 T0719-2011).

**Figure 19:**
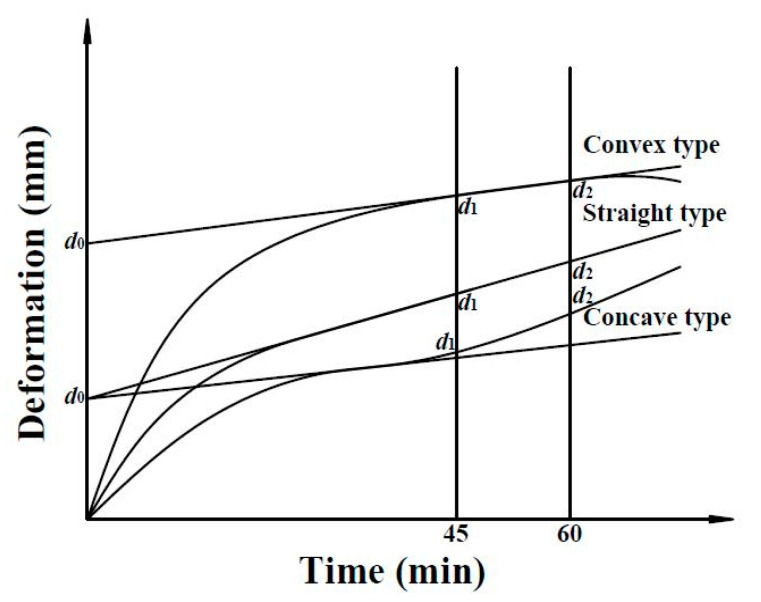
Deformation curve recorded during the rutting test (JTG E20 T0719-2011).

Finally, the rutting resistance of asphalt mixtures was evaluated with the rutting test (JTG E20 T0719-2011) [40]. The test is carried out on a slab specimen of asphalt mixture compacted into a square steel mold (300 mm × 300 mm × 50 mm) and conditioned at 60 ± 1 °C for at least 5 h. Then, a solid rubber wheel with a pressure of 0.7 ± 0.05 MPa was repeatedly passed over the specimen for 1 h or until the maximum deformation reached 25 mm. Three samples were prepared and tested. Figure 18 illustrates the slab mixture and the testing device used for performing the rutting test. During the test, temperature and the evolution of the curve deformation versus time were recorded, as shown in Figure 19.

To calculate the dynamic stability (*DS*), the rutting values *d*_1_ (mm) and *d*_2_ (mm) at *t*_1_ = 45 min and *t*_2_ = 60 min were recorded, respectively. When the increment in deformation rapidly reached a value higher than 25 mm before the end of the test (60 min), then the *t*_1_ was measured in correspondence to the deformation of 15 mm, while *t*_2_ was associated with rutting equal to 25 mm. Hence, *DS* is calculated as follows:

(5) $$DS=\frac{(t_{2}-t_{1})\times N}{d_{2}-d_{1}}\times C_{1}\times C_{2}$$

where *DS* is the dynamic stability of the asphalt mixture (passes/mm); *N* is the number of passes (back and forward) per minute, which in the case of the present research was equal to 42 passes/min; and *C_1_* and *C_2_* are coefficients associated with the testing machine and the asphalt mixture specimen size. For the specific machine used during this study, *C_1_* = 1 while *C_2_* = 1 for a square slab specimen of 300 mm. Higher values of *DS* indicate better rutting resistance.

### 4.3. Experimental Results on Asphalt Mixture

#### 4.3.1. Marshall Stability and Flow Value

**Figure 20:**
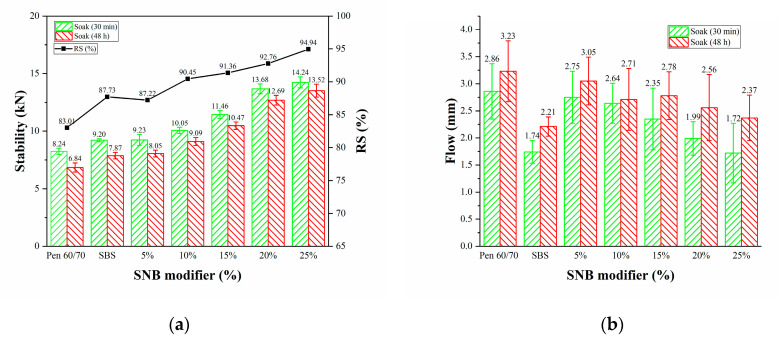
Marshall test: (**a**) stability and (**b**) flow (JTG E20 T0709-2011)

The Marshall stability and flow value (JTG E20 T0709-2011) [38] provide an empirical measure of the mixture performance for the Marshall mix design method. The Marshall stability consists of the maximum load (kN) applied to the cylindrical tested specimen with a displacement rate of 50 ± 5 mm/minute. The flow parameter is obtained by recording the vertical displacement (mm) obtained at the peak low (stability). In the present study, four samples were conditioned by soaking them in the water at 60 °C for 30 min, and four samples for 48 h, to estimate the response of the material to potential water damage. The residual Marshall stability, RS, defined as the ratio between the measurements obtained at 48 h and 30 min, was also computed. The results of the Marshall tests are reported in Table 14 and visualized in Figure 20.

Higher stability was observed for the SNB modified mixtures both for short- and long-term conditioning in water. An opposite trend was exhibited in the case of the flow values, suggesting the better overall performance of the SNB mixture at higher temperatures. A moderate increase in RS was also experienced for the SNB modified material with higher RS for the higher SNB contents. Figure 20 shows that the water damage resistance of all modified asphalts was greater than that of base asphalt, while the water damage resistance of SNB modified asphalts with more than 5 wt.% was greater than that of SBS modified asphalt. This indicates a better resistance to water damage for the modified mixtures, at least at a higher temperature.

#### 4.3.2. Indirect Tensile Strength and Moisture Susceptibility

**Figure 21:**
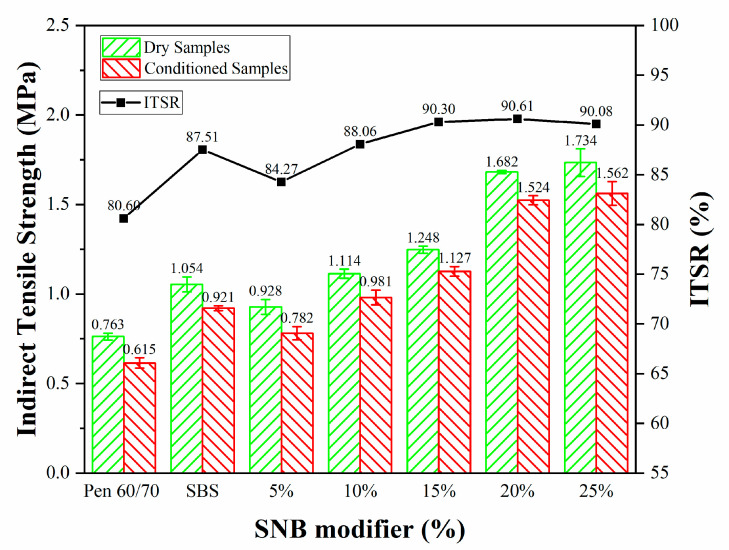
Results of the indirect tensile strength test (JTG E20 T0729-2011).

The ITS test and the indirect tensile strength ratio (ITSR) (JTG E20 T0729-2011) [39] were used to address the material behavior when subjected to one freeze–thaw cycle, and hence to estimate potential moisture damage. Table 15 and Figure 21 present the ITS values of test mixtures before and after freeze–thaw conditioning.

The results of the ITS tests revealed overall higher strength for the SNB mixture, both in the case of dry and conditioned samples which were exposed to a freeze–thaw cycle, compared with base asphalt. The water damage resistance of SNB modified asphalts with more than 5 wt.% content was greater than that of SBS modified asphalt. This appears to agree with the Marshall test. Additionally, the ITSR value tended to increase with higher amounts of SNB, suggesting a positive impact on the overall strength of the material compared to the original Pen60/70 mixture.

#### 4.3.3. Rutting Resistance

The rutting test (JTG E20 T0719-2011) [40] was used to evaluate the resistance to permanent deformation of the virgin, SBS, and modified mixture by determining the dynamic stability (DS). The values obtained from the rutting test are summarized in Table 16.

A significantly much higher DS (285%) was exhibited for the SNB mixture when compared to the Pen60/70 material. This provides evidence of a superior performance against the rutting of the mixtures designed with SNB modifier. Such behavior may be associated with the presence of a harder binder/mastic phase in the latter type of material capable of extending the resistance to permanent deformations. It was noticed that the rutting resistance of SNB modified asphalt with the SNB content higher than 5% was better than that of SBS modified asphalt, which is consistent with the previous MSCR and ZSV tests.

## 5. Discussions and Conclusions

In this paper, the possibility of incorporating Selenice natural bitumen (SNB), a natural harder asphalt binder, in the mix design of asphalt mixture was experimentally evaluated to benefit from the peculiar characteristics of such material. The investigation was initially devoted to determining the physical properties of the SNB material, and the conventional and rheological characteristics of binder blends prepared with a Pen 60/70 binder, an SBS modified binder and different contents of SNB. Next, an exploratory study was performed to address basic mixture properties, such as Marshall stability, indirect tensile strength, and rutting resistance. Based on the results obtained in the present research, a synthetic discussion is presented in the following subsection.

### 5.1. Discussions

At the binder level, the addition of SNB resulted in decreased penetration, in which the penetration of 25 wt.% SNB modified asphalt was only one-third of that of base asphalt, which is related to higher softening point and viscosity. However, ductility and BBR tests showed that the addition of SNB reduced the low temperature performance of asphalt. These results of penetration, softening point, viscosity, BBR, and ductility are consistent with those of XRA, QRA or BRA modified asphalt results [17,18,21,22,23,24]. In addition, the incorporation of SNB greatly improved the rutting factor, J_nr_, and zero shear viscosity of asphalt, indicating the superior high temperature performance of asphalt, which is consistent with the results of other natural modified asphalt [17,18,21]. An overall increase in stiffness was experienced for a higher amount of SNB, with a consequently better performance against rutting. At present, there are very few studies concerning the fatigue performance of natural modified asphalt. A considerable enhancement of fatigue behavior can be observed for a higher content of SNB, while this induces a potential decay of the response against low temperature cracking with a material presenting reduced relaxation capabilities. However, when the SNB content is higher than 15%, the fatigue performance of the SNB modified asphalt binder decreases. Therefore, the content of the SNB modifier should be limited.

At the mixture level, the incorporation of SNB material corresponds to an enhanced Marshall stability and better indirect tensile strength, which all meet the limitations of the standard. The values of RS and ITSR increased with the increase in SNB content, which is consistent with Zeng’s research results using European rock asphalt and AC-20C grading [66]. However, for the same amount of modifier, the values of RS and ISTR were lower than those of Zeng’s research [66]. In addition, significantly better rutting resistance was obtained in comparison to conventional mixtures, confirming the experimental results at the binder level. When the content of rock asphalt was more than 10%, the dynamic stability of rock asphalt was more than twice that of base asphalt.

MSCR, ZSV, and rutting test results showed that the high temperature performance of 5 wt.% SNB modified asphalt binder is worse than that of SBS modified asphalt, whereas with more than 5 wt.% SNB content, it showed better performance than that of SBS modified asphalt. This trend is not in agreement with the results of the rutting factor test, further proving its limitations. In addition, with the increase in SNB content, the difference of zero shear viscosity of SNB modified asphalt became larger. For example, the zero-shear viscosity of 25 wt.% SNB modified asphalt was more than twice that of 20 wt.% SNB modified asphalt. The ZSV test is capable of better discriminating between the high temperature performance of modified asphalt with a high content of SNB.

FM tests showed that after dissolving, the size of the SNB modifier became smaller and the distribution of SNB modifier was uneven, exhibiting three forms, granular, agglomerated, and flocculent properties. The results of the FTIR test showed that the modification mechanism of SNB was mainly related to the enhancement of hydrogen bonds and Van der Waals forces caused by S=O and C=O, and the stress concentration caused by silica particles. This led to an increase in softening point and rotational viscosity, whereas a decrease in ductility was also observed. GPC test results showed that the incorporation of an SNB modifier reduced the proportion of middle molecule size components and increased the proportion of large molecule size components. This resulted in increasing the softening point and rotational viscosity, while decreasing penetration.

According to the softening point difference test, the softening point difference of 25 wt.% is greater than the 2.5 °C requirement of the specification [51]. According to the BBR test, the low PG grade of 25 wt.% SNB modified asphalt was higher than that of the virgin, SBS, and other SNB modified asphalt binders. The LAS test revealed that 15 wt.% SNB modified asphalt had the best fatigue life and performance, whereas 25 wt.% SNB modified asphalt showed the lowest values compared with other SNB modified asphalts. From the mixture tests, the dynamic stability of 5 wt.% was less than 3000 passes/mm, as required by the specification [51], whereas the high temperature performance and water damage resistance of other SNB modified asphalt were relatively high. Therefore, based on all asphalt and asphalt mixture tests, the recommended dosage of the SNB modifier is 10–20 wt.%, and the optimal dosage is 15 wt.%.

### 5.2. Conclusions

The following conclusions can be drawn based on the above discussion:

(1) At binder level, the addition of SNB improves the high temperature performance of asphalt, and reduces the low temperature performance and storage stability. The fatigue performance of SNB modified asphalt first increases and then decreases with the increase in SNB content.
(2) At mixture level, the addition of SNB improves the water damage resistance and rutting resistance of the asphalt mixture.
(3) MSCR, ZSV, and rutting test results showed well and consistent high temperature performance; however, the rutting factor test has some limitations.
(4) FM, FTIR and GPC test results clearly showed the chemical properties and modification mechanism of SNB modified asphalt.
(5) According to all binder and mixture tests, the optimal dosage of the SNB modifier is 15 wt.% and the recommended dosage is 10–20 wt.%.

This paper deeply explored the modification mechanism of SNB, as well as provides comprehensive and systematic guidance for the use of SNB, including the physical, conventional, chemical, rheological, and mechanical performance of both SNB modified asphalt and its corresponding mixture. In this sense, contemporary research has shown the possibility of combining asphalt modified with natural bitumen, SBS and plasticizer to mitigate the potentially negative effect observed at low temperature. This represents the objective of a follow-up investigation.

## Figures and Tables

**Table 1:** Initial characterization of the asphalt binder.

Test	Result	Specification
Penetration (25 °C), 0.1 mm	63.5	JTG E20 T0604-2011
Penetration Index	−1.37	JTG E20 T0604-2011
Softening point (R&B), °C	48.9	JTG E20 T0606-2011
Ductility (15 °C), cm	>150	JTG E20 T0605-2011
Ductility (10 °C), cm	31.2	JTG E20 T0605-2011
Kinematic Viscosity (60 °C), Pa.s	208	JTG E20 T0619-2011
Wax content, %	<2.1	JTG E20 T0615-2011
Solubility in Trichloroethylene	>99.5	JTG E20 T0607-2011
Flash point, °C	335	JTG E20 T0611-2011

**Table 2:** Experimental program.

Properties	Characteristic or Performance	Tests	Aging Level	Specification
Physical	Ash Content	Mineral Matter or Ash in Asphalt Materials	unaged	JTG E20 T0614-2011
	Asphalt Binder Content	Determining the Asphalt Binder Content of HMA by the Ignition	unaged	JTG E20 T0735-2011
	Sieve Size	Sieve Analysis of Fine and Coarse Aggregates	after ignition	JTG E20 T0327-2011
Conventional	Penetration	Penetration	unaged, RTFOT	JTG E20 T0604-2011
	Softening point	Ring and Ball	unaged, RTFOT	JTG E20 T0606-2011
	Ductility	Ductility	unaged, RTFOT	JTG E20 T0605-2011
	Storage Stability	Softening Point Difference	unaged	JTG E20 T0661-2011
Rheological	Overall behavior	Temperature Frequency sweep	RTFOT	N/A
	High-temperature	Temperature sweep	unaged, RTFOT	JTG E20 T0628-2011
		MSCR	RTFOT	AASHTO T350-2019
		Zero Shear Viscosity	unaged	N/A
	Fatigue	LAS	RTFOT + PAV	AASHTO TP101-2014
	Low-temperature	BBR	RTFOT + PAV	JTG E20 T0627-2011
	Workability	Rotational Viscosity	unaged	JTG E20 T0625-2011
Mechanism investigation	Morphology	FM	unaged	N/A
	Chemical properties	FTIR	unaged	N/A
	Molecular composition	GPC	unaged	N/A

Abbreviations: hot mix asphalt, HMA; rolling thin-film oven test, RTFOT; multiple stress creep recovery, MSCR; linear amplitude sweep, LAS; pressure aging vessel, PAV; bending beam rheometer, BBR; Fluorescence microscopy, FM; Fourier-transform infrared, FTIR; Gel permeation chromatography, GPC.

**Table 3:** Gradation of the SNB particles after ignition.

Sieve (mm)	1.18	0.6	0.3	0.15	0.075	<0.075
Passing	100.00%	99.71%	84.57%	51.29%	29.57%	0.00%

**Table 4:** Parameters of the model of the master curves of G* at reference temperature 60 °C.

Parameters	WLF Formula		Sigmoidal Function				
	*C_1_* (-)	*C_2_* (-)	*δ* (-)	*α* (-)	*β* (-)	*γ* (-)	R^2^ @ |G*|
Pen60/70	13.88	191.7	−3.641	9.189	0.2605	−0.4194	0.9988
SBS	12.09	173.9	−3.275	8.831	0.1876	−0.3877	0.9987
+5% SNB	13.89	188.2	−3.913	9.494	−0.01747	−0.3905	0.9991
+10% SNB	15.73	207.2	−4.072	9.701	−0.09866	−0.3776	0.9989
+15% SNB	16.68	213.7	−4.261	10.020	−0.2106	−0.3540	0.9991
+20% SNB	16.92	210.8	−4.108	9.851	−0.2840	−0.3488	0.9990
+25% SNB	18.52	227.5	−4.478	10.340	−0.3728	−0.3264	0.9993

**Table 5:** Multiple Stress Creep Recovery (MSCR) results at 64 °C (AASHTO T350, 2019).

Binder Type	J_nr_			Percent Recovery R (%)		Traffic Level
	0.1 kPa	3.2 kPa	J_nr-diff_ (%)	0.1 kPa	3.2 kPa	
Pen 60/70	1.566 ± 0.152	1.715 ± 0.135	9.5 ± 0.3	3.5 ± 0.4	1.2 ± 0.2	H ^1^
SBS	0.788 ± 0.250	1.111 ± 0.294	42.2 ± 7.7	35.3 ± 4.0	12.2 ± 1.1	H ^1^
+5% SNB	1.459 ± 0.090	1.623 ± 0.102	11.3 ± 0.2	4.8 ± 0.2	2.0 ± 0.2	H ^1^
+10% SNB	0.888 ± 0.018	1.012 ± 0.008	14.0 ± 1.3	8.8 ± 0.3	4.3 ± 0.2	H ^1^
+15% SNB	0.454 ± 0.072	0.511 ± 0.083	12.6 ± 0.4	14.8 ± 1.8	10.3 ± 1.8	V ^2^
+20% SNB	0.376 ± 0.013	0.414 ± 0.021	10.0 ± 1.2	17.8 ± 0.8	13.2 ± 1.5	E ^3^
+25% SNB	0.128 ± 0.020	0.145 ± 0.026	13.0 ± 2.1	30.7 ± 2.3	26.2 ± 2.5	E ^3^

The numbers after “±” are standard deviations. Standard Designation “S” in most typical situations will be for traffic levels fewer than 10 million Equivalent Single Axle Loads (ESALs) and more than the standard traffic speed (>70 km/h). ^1^ High Designation “H” in most situations will be for traffic levels of 10 to 30 million ESALs or slow-moving traffic (20 to 70 km/h). ^2^ Very High Designation “V” in most situations will be for traffic levels of greater than 30 million ESALs or standing traffic (< 20 km/h). ^3^ Extremely High Designation “E” in most situations will be for traffic levels of greater than 30 million ESALs and standing traffic (< 20 km/h) such as toll plazas or port facilities.

**Table 7:** Linear amplitude sweep (LAS) test results (AASHTO TP101).

Binder Type	Strain Level	Fatigue Life (Cycles)
		Average Value
Pen 60/70	2.5%	23,126 ± 386
	5.0%	2457 ± 102
SBS	2.5%	34,881 ± 3768
	5.0%	3945 ± 311
+5% SNB	2.5%	29,652 ± 1024
	5.0%	2683 ± 86
+10% SNB	2.5%	36,215 ± 967
	5.0%	2751 ± 536
+15% SNB	2.5%	53,648 ± 3245
	5.0%	2803 ± 607
+20% SNB	2.5%	32,015 ± 4211
	5.0%	1637 ± 652
+25% SNB	2.5%	26,547 ± 3327
	5.0%	688 ± 37

The numbers after “±” are standard deviations.

**Table 8:** Bending beam rheometer (BBR) test results (JTG E20 T0627-2011).

**Binder Type**	**−18 °C**		**−12 °C**	
	**S (t = 60 s) (MPa)**	***m*-Value**	**S (t = 60 s) (MPa)**	***m*-Value**
Pen 60/70	373	0.246	222	0.316
SBS	381	0.230	209	0.307
+5% SNB	474	0.233	272	0.286
+10% SNB	490	0.240	274	0.292
+15% SNB	563	0.221	282	0.286
+20% SNB	596	0.210	346	0.256
+25% SNB	609	0.202	395	0.245
**Binder Type**	**−6 °C**		**0 °C**	
	**S (t = 60 s) (MPa)**	***m*-Value**	**S (t = 60 s) (MPa)**	***m*-Value**
Pen 60/70	121	0.386	/	/
SBS	101	0.381	/	/
+5% SNB	127	0.359	58.7	0.443
+10% SNB	140	0.359	71.6	0.417
+15% SNB	145	0.338	88.5	0.382
+20% SNB	203	0.301	100	0.361
+25% SNB	222	0.285	113	0.341

**Table 9:** SMA-10 mixing temperature based on the rotational viscosity test.

Binder Type	Temperature (°C)
Pen 60/70	160.0 ± 1.6
SBS	165.8 ± 1.3
+5% SNB	163.3 ± 1.3
+10% SNB	165.3 ± 1.2
+15% SNB	167.9 ± 1.0
+20% SNB	168.2 ± 0.7
+25% SNB	168.7 ± 0.5

**Table 10:** The ratio of large molecule size (LMS), medium molecule size (MMS), and small molecule size (SMS).

Sample	LMS	MMS	SMS
Pen60/70	11.3%	54.2%	34.5%
+5% SNB	12.6%	53.0%	34.4%
+10% SNB	13.7%	52.7%	33.6%
+15% SNB	14.3%	52.0%	33.7%
+20% SNB	14.8%	51.0%	34.2%
+25% SNB	16.0%	50.8%	33.2%

**Table 11:** GPC parameters.

Sample	Mp (g/mol)	Mn (g/mol)	Mw (g/mol)	Mz (g/mol)	PDI (-)
Pen60/70	875 ± 3	604 ± 13	1521 ± 50	4009 ± 57	2.51707 ± 0.03008
+5% SNB	873 ± 1	593 ± 17	1596 ± 2	4551 ± 3	2.69171 ± 0.08061
+10% SNB	862 ± 9	603 ± 2	1674 ± 32	4778 ± 117	2.77770 ± 0.06259
+15% SNB	877 ± 13	603 ± 3	1707 ± 33	4996 ± 118	2.83018 ± 0.06839
+20% SNB	871 ± 4	605 ± 12	1787 ± 8	5400 ± 47	2.95689 ± 0.07284
+25% SNB	871 ± 4	602 ± 16	1828 ± 54	5504 ± 2	3.03641 ± 0.01081

The numbers after “±” are standard deviations.

**Table 12:** SMA-10 gradation used in this research (JTG F40-2004).

BS Sieve Size	Design Data (%)	Passing Requirement (%)
13.2 mm	100.0	100
9.5 mm	96.6	90–100
4.75 mm	46.0	28–60
2.36 mm	25.6	20–32
1.18 mm	21.0	14–26
0.6 mm	17.1	12–22
0.3 mm	14.5	10–18
0.15 mm	13.4	9–16
0.075 mm	11.6	8–13

**Table 13:** Experimental program.

Characteristic or Performance	Tests	Specification	Limit
Water Damage Resistance	Marshall test	JTG E20 T0709-2011	≥80%
	Indirect Tensile Strength test	JTG E20 T0729-2011	≥80%
High Temperature Stability	Rutting test	JTG E20 T0719-2011	≥3000 (passes/mm)

**Table 14:** Marshall test: experimental results for virgin, SBS, and SNB modified mixtures (JTG E20 T0709-2011).

Binder Type	Stability (kN)			Flow (mm)	
	Soaking Time (30 min)	Soaking Time (48 h)	RS (%)	Soaking Time (30 min)	Soaking Time (48 h)
Pen60/70	8.24 ± 0.27	6.84 ± 0.40	83.01%	2.86 ± 0.51	3.23 ± 0.56
SBS	9.20 ± 0.13	7.87 ± 0.28	87.73%	1.74 ± 0.21	2.21 ± 0.18
+5%SNB	9.23 ± 0.47	8.05 ± 0.29	87.22%	2.75 ± 0.48	3.05 ± 0.44
+10%SNB	10.05 ± 0.27	9.09 ± 0.35	90.45%	2.64 ± 0.37	2.71 ± 0.57
+15%SNB	11.46 ± 0.34	10.47 ± 0.33	91.36%	2.35 ± 0.57	2.78 ± 0.44
+20%SNB	13.68 ± 0.42	12.69 ± 0.42	92.76%	1.99 ± 0.31	2.56 ± 0.61
+25%SNB	14.24 ± 0.48	13.52 ± 0.56	94.94%	1.72 ± 0.55	2.37 ± 0.42

The numbers after “±” are standard deviations.

**Table 15:** Results of the ITS test on virgin, SBS, and SNB modified mixtures (JTG E20 T0729-2011).

Binder Type	Dry Samples (MPa)	Conditioned Samples (MPa)	ITSR (%)
Pen60/70	0.763 ± 0.019	0.615 ± 0.029	80.60%
SBS	1.054 ± 0.042	0.921 ± 0.014	87.51%
+5%SNB	0.928 ± 0.042	0.782 ± 0.037	84.27%
+10%SNB	1.114 ± 0.025	0.981 ± 0.041	88.06%
+15%SNB	1.248 ± 0.020	1.127 ± 0.027	90.30%
+20%SNB	1.682 ± 0.009	1.524 ± 0.025	90.61%
+25%SNB	1.734 ± 0.077	1.562 ± 0.066	90.08%

The numbers after “±” are standard deviations.

**Table 16:** Results of the rutting test on virgin, SBS, and SNB modified mixtures (JTG E20 T0719-2011).

Binder Type	Dynamic Stability (DS) (passes/mm)
Pen60/70	1636 ± 204
SBS	3289 ± 254
+5%SNB	2558 ± 345
+10%SNB	3723 ± 530
+15%SNB	4531 ± 365
+20%SNB	5124 ± 412
+25%SNB	5767 ± 607

The numbers after “±” are standard deviations.

**Table 6:** Parameters of the simplified Cross/Sybilski model of the zero-shear viscosity (ZSV).

Parameters	*K*	*m*	*η*_0_	R^2^ @ η*
Pen60/70	0.0002888	0.4712	311.3	0.9987
SBS	0.0260400	0.2082	658.3	0.9915
+5% SNB	0.0009205	0.4720	522.2	0.9982
+10% SNB	0.0020360	0.2561	961.6	0.9906
+15% SNB	0.0102000	0.3020	1739	0.9964
+20% SNB	0.0169400	0.3714	2613	0.9905
+25% SNB	0.0686300	0.3455	5321	0.9969

The numbers after “±” are standard deviations.

The numbers after “±” are standard deviations.

## Data Availability

The data presented in this study are available on request from the corresponding author.

## Abbreviations

selenice natural bitumen	SNB
reclaimed asphalt pavement	RAP
high modulus asphalt	HiMA
Xinjiang rock asphalt	XRA
Qingchuan rock asphalt	QRA
Buton rock asphalt	BRA
stone mastic asphalt	SMA
penetration grade of 60/70	Pen 60/70
nominal maximum aggregate size	NMAS
multiple stress creep recovery	MSCR
zero shear viscosity	ZSV
linear amplitude sweep	LAS
bending beam rheometer	BBR
fluorescence microscopy	FM
Fourier-transform infrared	FTIR
gel permeation chromatography	GPC
indirect tensile strength	ITS
hot mix asphalt	HMA
softening point difference	SPD
dynamic shear rheometer	DSR
rolling thin-film oven test	RTFOT
viscoelastic continuum damage	VECD
pressure aging vessel	PAV
time–temperature superposition principle	TTSP
Williams–Landel–Ferry	WLF
average percent recovery	R
non-recoverable creep compliance	J_nr_
stress sensitivity	J_nr-diff_
performance grade	PG
large molecule size	LMS
middle molecule size	MMS
small molecule size	SMS
peak molecular weight	Mp
number average molecular weight	Mn
weight average molecular weight	Mw
Z-average molecular weight	Mz
polydispersity index	PDI
dynamic stability	DS
residual Marshall stability	RS
indirect tensile strength ratio	ITSR
